# Scenarios for Ecodesign in loudspeaker’s motor

**DOI:** 10.1038/s41598-022-24042-7

**Published:** 2022-11-14

**Authors:** Allan Di Cunto D’Avila de Almeida, Ivan Aritz Aldaya Garde, Mirian Paula dos Santos, Rafael Abrantes Penchel, Lúcio Cardozo Filho, José Augusto de Oliveira

**Affiliations:** Center for Advanced and Sustainable Technologies - CAST, São Paulo State University (UNESP), Av. Profa. Isette Corrêa Fontão, 505, São João da Boa Vista, São Paulo 13876-750 Brazil

**Keywords:** Environmental impact, Structural materials, Design, synthesis and processing

## Abstract

The worldwide loudspeaker market follows the growing tendency of electronic entertainment technologies both in quantity and variety. Consequently, the environmental impacts caused during the life cycle of loudspeakers increase in the same proportion, going in the opposite direction to what is determined by world environmental laws and regulations and global market tendencies. Even so, the environmental performance of this type of product is not considered in the decision-making process for technological updates in loudspeaker design. In this sense, Ecodesign is the most adequate Life Cycle Engineering tool applied in the design of a product since the environmental performance is considered throughout the different design stages. However, the feasibility of Ecodesign in products requiring complex production chains relies on splitting the product into subsystems and components. Thus, the present work focuses on evaluating the environmental performance of a classic loudspeaker motor, which is composed of a magnet, coil, and coil former. Eight raw material substitution scenarios are proposed and analyzed, which allowed the proposal identification with the best environmental performance within the current technologies. This represents an initial step toward the complete Ecodesign of a loudspeaker and sets the procedure to be followed with the other constitutive parts.

## Introduction

The Compound Annual Growth Rate (CAGR) of the loudspeaker global market is increasing, and it is expected to grow at a rate of 7.3% until 2028 (FMI, 2018). Of this amount, North America and Southeast Asia have 40% of the market share. Consequently, unless environmental aspects are considered during the design of this product, its environmental impacts will grow inexorably^1,2^. This tendency contradicts the goals of sustainable development (SDG) of Agenda 21 of the United Nations (UN) and the Circular Economy, which is the current model of the global economy^3^.

The objective of most organizations is to satisfy the implicit needs of the customer, and there is a need to restructure the product in order to sustain and shape the existing relationship between the manufacturer and end user. Sustainability is the balance or integration of environmental, social, and economic issues; in 1987, World Commission on Environment and Development (WCED) defined it as development that meets the needs of the present without compromising the ability of future generations to meet their own needs^4^. In this context, there are several laws and regulations worldwide that encourage the adequacy of businesses to the principles of the Circular Economy. For instance, in the United States of America, there are laws and regulations provided for the Resource Conservation and Recovery Act (RCRA)^5^. The European legislation counts with the Restriction of Certain Hazardous Substances (RoHs)^6^, which limits the use of some substances in product manufacturing processes, and the Waste Electrical and Electronic Equipment (WEEE)^7^ that imposes obligations on organizations, containing the rules applicable to electronic waste. In general, this directive encourages and defines specific criteria for the collection, handling, and recycling of electrical and electronic waste^1,4^. In Brazil, for example, the Law no. 12.305/10^8^, which institutes the National Solid Waste Policy (PNRS), provides prevention and reduction of waste generation, a proposal for the practice of sustainable consumption habits, and a set of instruments to increase recycling and reuse of solid waste.

In addition, some standards have been proposed to encourage the adaptation of organizations to the context of improving environmental aspects related to their activities. Some remarkable examples within the ISO 14000 family are ISO 14001, which provides guidelines for an Environmental Management System, ISO 14040 (2009)^9^ and ISO 14044 (2009)^10^ that contemplate practices for the Life Cycle Assessment (LCA) of products, and ISO 14006 (2020)^11^, which addresses the implementation of Ecodesign or Design for Environment. These standards include plans for decision-making processes that contribute to the prevention of environmental impacts such as contamination of soil, water, and air^9,10^.

Regarding product design, considering the entire life cycle perspective, Ecodesign stands out as one of the main techniques for the productive sector in search of the Circular Economy^12,13^. However, in products with complex manufacturing chains already established in the market, such as the case loudspeakers, changes in the entire product design are difficult to apply in practice, as they demand complex costs and time-consuming evaluations. Thus, stratifying the product (system) into subsystems and components can be a feasible root to applying Ecodesign in this kind of product. In addition, the approach mentioned above can be further improved with a previous application of LCA to identify environmental hotspots and classify priorities for applying changes in the product design aiming at Ecodesign^13^.

The LCA also applies to decision-making processes during the product engineering stage, proposing the use of materials that are less aggressive to the environment and rationalizing and optimizing the use of energy and raw materials. In addition, based on LCA results, the engineers can design products with an extended useful life, facilitating their disassembly for the use of their components and enabling the recycling of their materials^14^.

In Operations Management, LCA can contribute to defining the choice of production resources, in addition to how activities related to the product manufacture or the provision of services will be integrated. Based on the results of LCA, the planning of resource use, material needs, product development, and production control are more efficiently prepared. Therefore, it is important to note that LCA is not limited to modifying product design, but it extends to their production processes. If a process is not assessed positively from the view of sustainability, for example, because it demands a large number of materials or energy or due to the generation of excess waste, this process should be the object of studies and improvements^14^.

Compliance with environmental laws is not only a matter of obligation. The reputation of an organization is strongly linked to the way it deals with the environmental aspects of its products, services, and processes. An organization must have a good reputation not only to consolidate its image but also to take advantage of business opportunities since many companies require their partners to have certifications that prove their compliance with environmental standards^15^. The LCA of products makes it possible to find alternative sources of raw material and energy, develop processes that require fewer inputs and generate less waste, identify the possibility of reusing by-products or parts of the finished product, and give an adequate destination to the product after its disposal by the consumer.

### General loudspeaker characterization and assembly materials

Ideally, the consumer should hear a sound like that planned by whoever recorded it. With this goal, the best acoustic speakers recreate the sound as close as possible to the original. One of the most important and informative performance metrics is the loudspeaker’s frequency response. The frequency response can be split into amplitude and phase, which together completely describe the linear behavior of the system^16,17^. The main goal is to get a loudspeaker that can accurately reproduce the frequencies of the whole human hearing spectrum. In this way, the more uniform the amplitude and the phase response over the whole operation bandwidth is, the better the quality of the loudspeaker. In a frequency response graph, it is desirable to see a straight line rather than a line with peaks and valleys. Given an ideal signal from an ideal amplifier and audio source, variations in the flat frequency response can often be attributed to its construction processes and materials used, which can vary significantly. For example, the propagation cone (a loudspeaker component) can be made of paper, aluminum (Al), polypropylene, or fiberglass/ceramic polymer.

Regarding their operation principle, most speakers operate similarly: on the back of the loudspeaker, generally, a circular magnet is held firmly in place with a rigid frame. A coil is placed around the magnet. However, in contrast to the magnet, the coil is attached to a moving piece; as the loudspeaker is supplied with a voltage, changes in the electric field cause a copper (Cu) coil inside the magnet to move. Attached to this there is a membrane, usually made of paper or plastic, which moves together back and forth, displacing air, thus creating sound waves. When an electrical current flows in one direction, the membrane moves away from the magnet, and when it flows in the other direction, the membrane moves closer. The current flow is changed from one side to the other, corresponding to the frequency induced. For low frequencies, this can be a few dozen times per second. For high frequencies, this happens up to 20,000 times or more per second. The size of a loudspeaker affects the range of audio frequencies it can produce, so a larger loudspeaker can move more air, but not quickly, making it better at producing lower frequencies. A smaller loudspeaker does not move as much air and consequently can move much faster, making it better to produce higher frequencies^16^.

### The environmental impacts of the loudspeaker and its product design phases for the Ecodesign

The importance of Ecodesign in product projects, such as the loudspeaker, and the application of LCA in product subsystems or components, for instance, in its motor, is clear. However, to the best of our knowledge, no report of empirical results that have assessed and identified the environmental impacts on the loudspeaker motor life cycle for future application of Ecodesign in this product can be found in the literature. Indeed, a literature survey reveals that most of the studies are limited to evaluating, either empirically or theoretically, the effects of noise pollution on people, as can be seen in^18–20^. These studies are applied to the loudspeaker use phase, with the acoustic bias to improve the product design. Some exceptions are^21,22^, where authors evaluated the environmental impacts of the neodymium magnet, comparing the environmental impacts of the virgin magnet with the recycled magnet. The authors concluded that the recycled neodymium magnet has a lower potential for environmental impacts than the virgin magnet. In particular^21^, highlights the following categories: Global Warming; Acidification; Human carcinogenic toxicity; Non-carcinogenic human toxicity; Human Health Particulate Air; Eutrophication; Depletion of the ozone layer; Ecotoxicity; and Smog.

To perform an Ecodesign of a loudspeaker, we adopted a divide-and-conquer approach, stratifying the whole product into smaller subsystems. Particularly, we focused on the loudspeaker’s motor. Once the object of study is delimited, LCA is applied to different proposed scenarios, aiming to support the engineering decision-making process and optimize resources to enable the practice of Ecodesign in this productive sector.

Thus, the present research proposes Ecodesign alternatives for loudspeaker motors based on the environmental performance of scenarios empirically assessed by LCA. The scenarios were generated considering different combinations of components already in use in the current market for loudspeaker motors.

The present work will be useful to support the decision-making process during the engineering of loudspeaker products and will cover a gap in the scientific literature on the subject. After this introduction, the article is structured as follows: In “Methods” section, the used research method is explained. The obtained results, including the generated scenarios, are presented and discussed in “Results and discussions” section. Finally, the conclusions are drawn in “Conclusion” section.

## Methods

**Figure 1:**
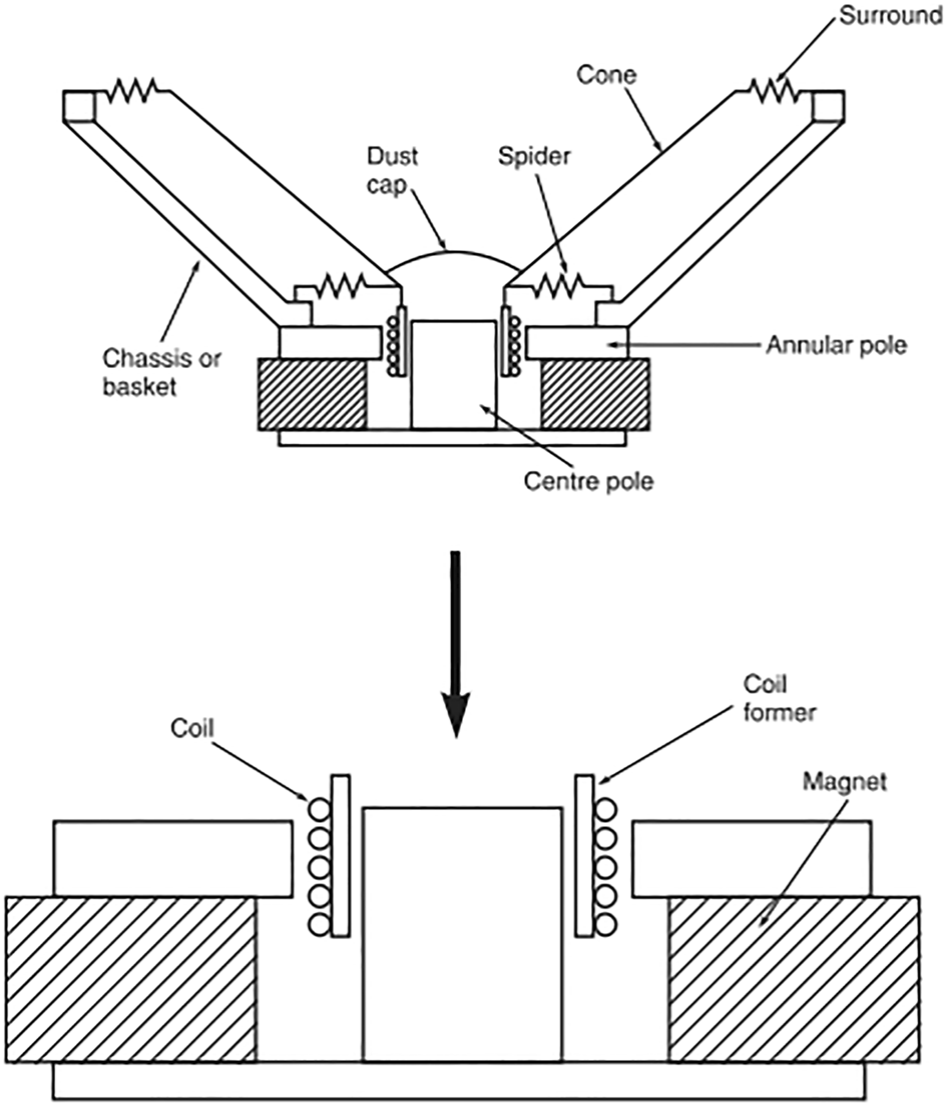
Structure drawing of a conventional speaker highlighting each component and zooming in on its motor set.

Loudspeakers have been designed and produced with permanent magnets for over 50 years^23^. The first evolution occurred when the motor magnet was replaced by an Alnico (aluminum/nickel/cobalt). Therefore, these loudspeakers were still quite long and complex and weighed devices. The first turning point was to decrease in height and size. So, it uses hard ferrite magnets^24^. Designs with ferrite magnets are inefficient as there is much flux leakage. On the other hand, ferrite magnets have economic advantages due to their price on the market. But, iron in such motors leads to several kinds of nonlinearities. These include, for example, the magnetic saturation of the iron and the variation of the coil inductance with its position, causing a reluctant effect^25^. The appearance of neodymium permanent magnets is the last step linked to the progress in permanent magnet materials. With such permanent magnets with Nd compound, the size and weight of the hole motor decreased dramatically^26^. In this way, modifying the permanent magnet with a smaller and lightweight is necessary for a new design of the entire loudspeaker motor (coil and coil former), making it possible to employ new materials and even biomaterials. Changes in the motor performance are also due to components’ mechanical tolerances, their influence is important to check to guarantee repeatability during production, and high product performances^27^. Due to the complex production chain of the product, comprising several production units in different parts of the globe, the object of this research was delimited to the loudspeaker motor, highlighted in Fig. 1, which is one of the most critical and differential parts of a loudspeaker.

There are many loudspeaker motor models widely used in the market that have similar functionalities and characteristics. We can roughly divide the motor in three main constitutive parts, meaning the magnet, the coil former, and the coil, which can be built employing different materials. Table 1 lists a possible configuration to baseline scenario, denominated from here on “Real product,” and presents another seven scenarios as Ecodesign propositions. For the sake of information confidence of the manufacturer, the name and description of the loudspeaker model were kept confidential, considering in this article as the Real product. The Real product configuration was adopted as the baseline scenario because it is the most used structure for loudspeaker motors in the market.

**Table 1:** Components of the models of loudspeakers motors.

Possible scenarios	Coil	Coil former	Magnet
1 (Real product)	Aluminum	Aluminum	Ferrite
2	Aluminum	Aluminum	Neodymium
3	Aluminum	Fiberglass	Ferrite
4	Aluminum	Fiberglass	Neodymium
5	Copper	Aluminum	Ferrite
6	Copper	Aluminum	Neodymium
7	Copper	Fiberglass	Ferrite
8	Copper	Fiberglass	Neodymium

**Figure 2:**
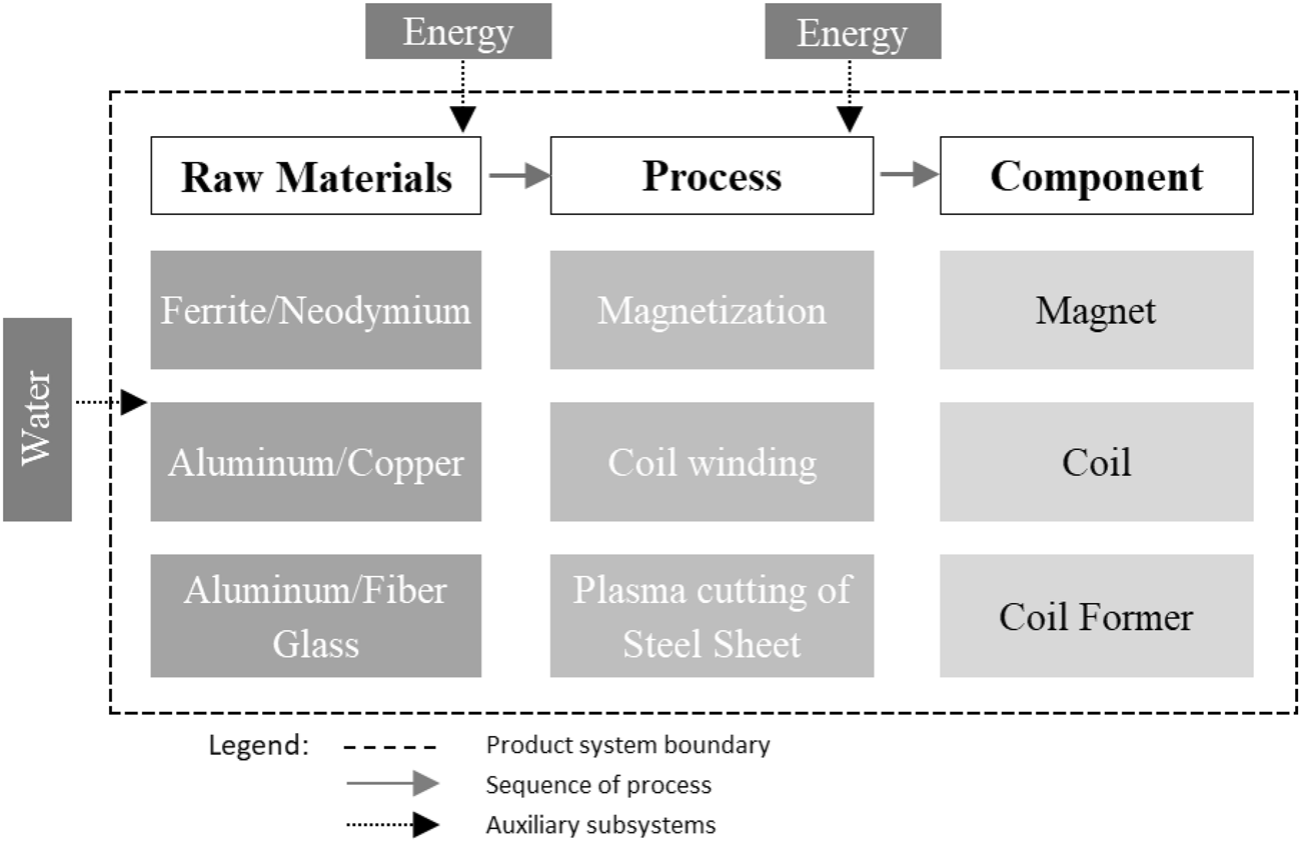
Grayscale diagram with three columns/steps with arrows signaling the inputs and outputs of the flow of materials, components, and processes in the production of a conventional loudspeaker.

Thus, the LCA was applied to each possible combination of the components of the motor models, with cradle-to-gate product systems, as shown in Fig. 2.

In Fig. 2, there are different components from those described in Table 1 because, in the product system, the other possible components were also considered for the combinations to generate the Ecodesign scenarios.

The material acquisition includes transport and displacement of the main raw materials of the loudspeaker. Most of the materials, such as aluminum and copper, for example, are supplied by regional companies. However, in the case of Nd, most of its extraction is concentrated in China^4,28^ and shipped to Brazil via maritime transport. Since material transport causes a smaller portion of overall product life cycle emissions, it needs to be considered in the analysis. The objective of the LCA is to evaluate the environmental impacts of 8 possible scenarios in the loudspeaker design, having as a comparison reference a project widely used in the market, entitled in this article as the Real product (baseline scenario), presented in Table 1. The scenarios (Ecodesign projects) studied have approximate functionalities and can be considered equivalent to standardizing a functional unit between them. To perform the LCA, the functional unit of 1 loudspeaker motor (Real product) of 960.927g was adopted. And the reference stream for this product is the production of 960.927g distributed among their three components, considered as the finished product.

The phases of LCA were conducted according to ISO 14040 (2009)^9^ and ISO 14044 (2009)^10^, using the software GaBi Student. The Life Cycle Inventory (LCI), as the second phase, was collected from loudspeaker datasheets. This is a secondary data collection method^29^. In the sequence, the next LCA phase is the Life Cycle Impact Assessment (LCIA). As a result of the modeling, data from four methodologies were obtained. Still, the analysis will focus on TRACI 2.1, as it has indicators mainly focused on the industrial area of North America and, therefore, are more suitable for the region of interest, i.e., Brazil, than the other methodologies, which are more oriented to Europe. The most relevant categories for the scenario of raw material extraction and loudspeaker production of the TRACI 2.1 methodology will be exemplified below^30^. So, in the same sense, the internal and external normalization were accomplished according to TRACI 2.1 methodology, considering the North American context. The internal normalization was carried out within the same scenario, where the total impact in each environmental impact category was evaluated, and the relative potential of each loudspeaker motor component for all the categories was calculated. The external normalization was performed according to:

1 $$\begin{aligned} NF_i = \frac{\sum {CF_{i,s}\cdot E_s}}{P} \end{aligned}$$

where:

- NF_i_ is the normalization factor (impact capita$^{-1}$ year$^{-1}$) for impact category *i*;
- CF_i_,_s_ is the characterization factor (impact kg$^{-1}$ emitted of a given substance s for impact category *i*;
- E_s_ is emissions of substances for a given geographical reference area (kg year$^{-1}$). In this study, we employed the US and US-CA populations; and
- P is the human population of the reference area (capita).

For more details of the normalization methodology, the reader is referred to^31,32^.

## Results and discussions

In this section, we first present a brief description of the potential environmental impacts listed in Table 2. The LCI results are shown in the Supplementary Material. Afterward, an internal normalization was carried out, showing the relative potentials of each component of the loudspeaker motor for each category of environmental impact. Then, a sensitivity analysis was performed, generating scenarios that represent options for the Ecodesign of the loudspeaker motor, together with an external standardization, considering the total impact by category in North America, which covers the most representative countries in the loudspeakers market, in addition to being the countries that are part of the LCIA TRACI 2.1 method.

**Table 2:** Life Cycle Assessment results of baseline scenario motor.

Impact category (TRACI 2.1)			Coil	Former coil	Magnet	Loudspeaker motor real product
Description	Acronym	Unit	Aluminum	Aluminum	Ferrite	(baseline scenario)
Global Warming Air	GW	kg CO_2_ eq.	7.34E−01	3.94E−02	7.71E+01	7.79E+01
Acidification	Ac	kg SO_2_ eq.	1.34E−03	6.93E−05	2.64E+01	2.64E+01
Eutrophication	Eu	kg N eq.	4.09E−05	2.25E−06	7.20E−02	7.20E−02
Ozone Depletion Air	OD	kg CFC 11 eq.	2.55E−09	− 8.76E−16	4.18E−06	4.18E−06
Ecotoxicity	Ec	CTUe	9.71E−03	5.05E−04	4.17E+02	4.17E+02
Human Health Particulate Air	HHP	kg PM_2.5_ eq.	1.92E−04	9.88E−06	2.50E-01	2.50E−01
Human toxicity, cancer	HTC	CTUh	1.99E−10	1.03E−11	1.99E−10	4.08E−10
Human toxicity, non-cancer	HTnC	CTUh	1.68E−08	8.74E−10	8.74E+03	8.74E+03
Smog Air	Smog	kg O_3_ eq.	1.56E−02	8.28E−04	2.00E−01	2.16E−01

To present the results in Table 2, it is first necessary to explain the causes and effects of the identified environmental impact categories will first be summarized in a general context. Then, the causes of these environmental impacts will be analyzed in the context of the loudspeaker motor.

According to^33^, Global Warming is mainly caused by the burning of fossil fuels, when the substances resulting from the burning are absorbed by infrared radiation and stabilize in the atmosphere (IPCC, 2007). This impact category is responsible for accelerated warming and sudden change in temperature on the globe.

Acidification is generally caused by the emission of gaseous, solid, or liquid pollutants, reaching the air, water, and soil, resulting mainly from activities and combustion in energy generation processes, whether electrical or thermal. In this case, the pollutants introduce or release hydrogen ions into the environment, and anions (that accompany hydrogen ions) are leached or washed out from the system^33^.

Eutrophication accounts for an excessive amount of nutrients in a medium. The main nutrients are based on Nitrogen, Phosphorus, and Potassium. According to^33^, Eutrophication can be caused especially by emissions into the air (e.g., nitrogen oxides from combustion processes), water (e.g., nitrogen in the aquatic environment originating from the use of fertilizers in agriculture), and in and over the soil (e.g., emissions of phosphorus leaching into the soil from agricultural sources).

Ozone layer depletion is caused especially by human-made emissions of halocarbons and gases at normal atmospheric temperatures, such as refrigerant substances, solvents, and foaming agents containing chlorine or bromine. In particular, refrigerant substances are especially harmful since they are still commonly used in heat cycles for enhanced efficiency, a reversible phase transition from liquid to gases^33^.

According to^33^, Ecotoxicity is caused by the emission of toxic substances into the biosphere that affects species of flora and fauna, causing toxicity in their species, which can be bioaccumulative. The Ecotoxicity is quantified in Comparative Toxic Units Ecotoxicity (CTUe).

The Human Health Particulate Air is associated with microparticles causing health problems. The microparticles have less than 10 micrometers in diameter and can get deep into the lungs, and some may even get into the bloodstream. Therefore, these particles may affect the human lungs and heart. People with heart or lung diseases, children, and older adults are the most likely to be affected by particle pollution exposure^33^.

Measured in Comparative Toxic Unit for human (CTUh). Human toxicity can be divided into two main categories depending on whether it is susceptible to causing cancer or not. The former Human toxicity is induced by chemical emitted substances that are ingested or inhaled by humans, and those substances can cause cancer. Human toxicity, non-cancer, is also caused by the ingestion or inhalation of chemical substances emitted into the environment by human activities, but which cause other harm to human beings, except for cancer^29^.

Finally, according to^29^, there is Tropospheric Ozone Formation, also called Smog formation or just Smog as used in this paper. At the ground level, for ozone, the same chemical reaction occurs between nitrogen oxides (NOx) and volatile organic compounds (VOCs) in the presence of sunlight. These gases are primarily generated by electric power utilities, industrial facilities, and combustion motors. The Smog causes various respiratory diseases, such as bronchitis, asthma, and emphysema. The ecological impacts include depletion of the same ecosystems^5^.

The productive chain of the loudspeaker motor is composed of magnet production, coil production, and coil former production. The main environmental aspects of these processes and their main environmental impacts will be presented and discussed below.

### Magnet production

The largest emission contributing to the global warming category is the CO_2_ eq. provided by the production step of the ferrite-base raw material employed to produce the magnet. In addition, the largest emission affecting human toxicity (carcinogenic agents) was arsenic (As) in the air, with the greatest impact also coming from incineration and solid waste disposal. For non-cancer agents, human toxicity responsible agent, Pb-contaminated water, had the greatest emission impact. Regarding respiratory effects, the biggest contributor was the beneficiation process to manufacture the ferrite magnet. In the Eutrophication category, the emission that had the greatest impact resulted from the phosphate distributed in the water and from the depletion of O_3_ or CH_4_ present in the air. In the literature, one sees burning of fossil fuels in industrial furnaces was the biggest contributor to the photochemical formation of atmospheric pollution (smog) category^21,22^.

For a ferrite loudspeaker magnetic assembly to perform the same efficiency as neodymium, approximately four times the mass of ferrite (to have the same magnetic flux) is needed^22^. In this analysis, neodymium was the material that contributed the least negatively, as all categories were on average 25% smaller than ferrite. It is noteworthy that Nd (magnet used in high-performance loudspeakers) has extraction and primary production concentrated in China since 1994, with the demand for its applications increasing since 1990 for both virgin and recycled material. The use of neodymium magnets in the global market represents 6,2%, whereas the largest use is for electric motors with 34%^22^.

### Coil production

After ferrite, aluminum predominates in most of the loudspeaker motor’s environmental impact categories. The data presented in Table 2 clearly reflect the difference in the environmental impacts of ore processing, considering the extraction, separation, and concentration of the desired minerals. In the global warming category, CO_2_ emission occurs mainly through the combustion of fossil fuels, which in the case of aluminum, after crushing by tractors and excavators in the bauxite extraction stage, various industrial processes with separation machinery are required, washing (large amounts of water), grinding and disposing of the tailings separated from the washed bauxite. Analyzing the aluminum inventory in Supplementary Material, the amounts of emissions of organic substances to air and water, we can observe that it presents values twice of the copper. Concerning copper, the only outlier and higher than aluminum was in the Ecotoxicity category, which can be explained by the high amount of particulate and heavy metal emissions into the air. This difference can be attributed to the different chemical processes involved in aluminum production. Thus, in loudspeakers, the choice of copper improves the performance from an environmental point of view. However, in relation to the material density, aluminum is preferable because a desirable characteristic of the coil is to be light so as not to influence the displacement of the set and, consequently, the acoustic quality.

### Coil former production

**Figure 3:**
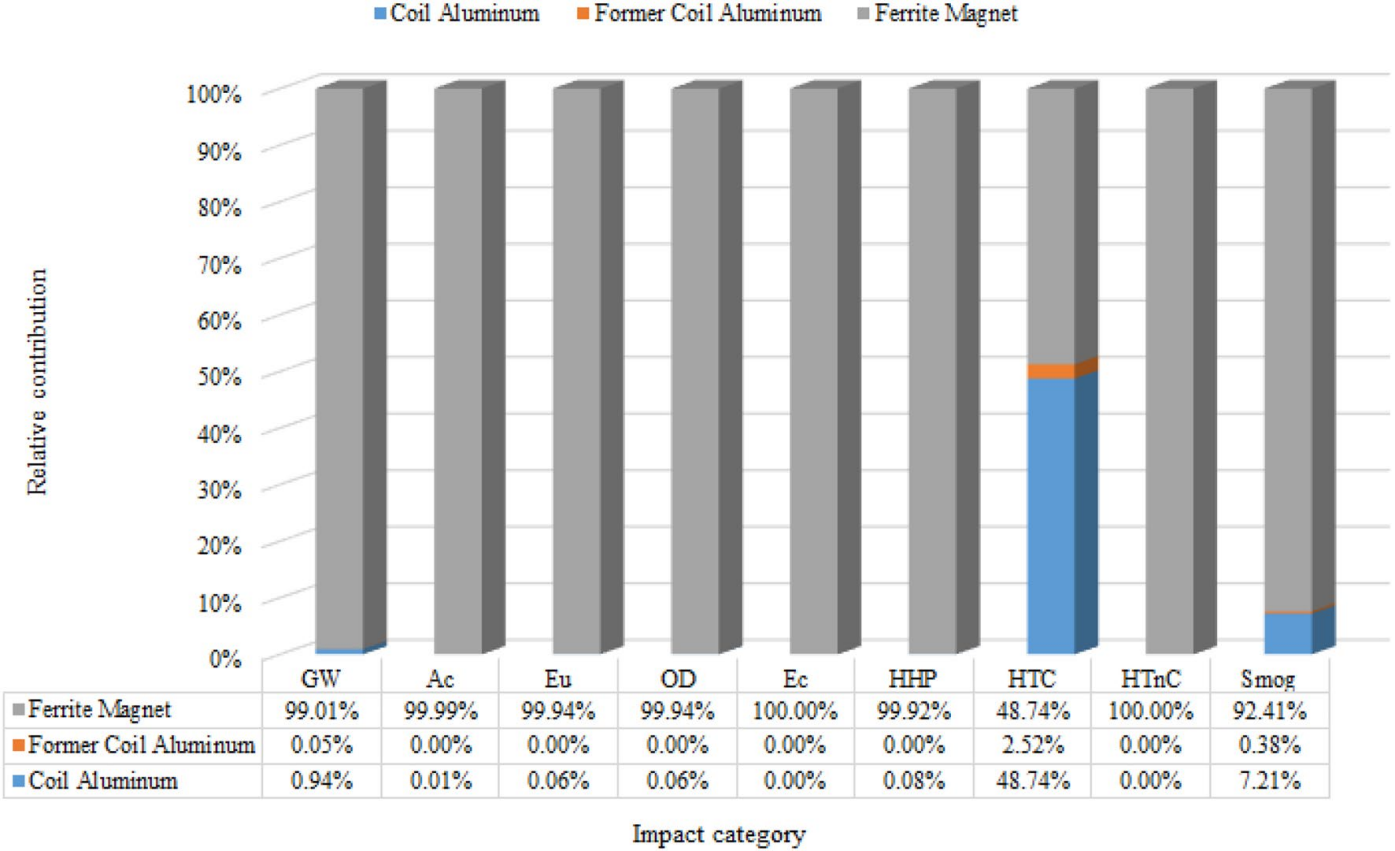
Bar graph with two axes and the three materials representing the relative contribution in percentage by impact category by categories, highlighting the HTC aluminum coil category with the highest percentage.

In^30^, the results of applying LCA to coil former production are described by^34^, comparing the life cycle impacts of the aluminum and copper coil form. For the coil form, the bauxite extraction process goes through the same procedure described in the production of aluminum coil, differentiating in the final step in which the aluminum sheets are formed and not billet as the one destined for the coil. The impact grades for fiberglass were better than aluminum. Its manufacturing process is simpler than aluminum, using smaller amounts of minerals in its composition and requiring less machinery. In the case of minerals, we can see in the inventory in Supplementary Material that the presence of Colemanite (Ca_2_B_6_O_11_.5H_2_O) and Dolomite (CaMg(CO_3_)_2_) are far from negligible. The O_3_ layer depletion value for both materials was negative, this value indicates that the analyzed alternative resulted positively for the impact category, and finding the root cause of this discrepancy is not part of the scope of the study, but it can be indicated for future in-depth study. The use of fiberglass in loudspeakers becomes interesting both in terms of environment and performance since fiberglass has better stiffness and operating temperature (before deforming) than aluminum. Figure 3 shows the relative contributions of environmental impacts of each component of Scenario 1 of the loudspeaker motor as a function of each assessed environmental impact category. It is important to mention that Scenario 1 is the most common one found in the loudspeaker industry, so it was considered a base scenario for comparing the other scenarios shown in Tables 1 and  3.

Considering ferrite as a magnetic component material, this component predominates in all categories of environmental impacts, and it is not possible to identify the contributions of potential environmental impacts from other components and materials in the loudspeaker motor. Thus, the premise is that aiming at the best environmental performance for this product, ferrite should be replaced by neodymium in all scenarios. Thus, scenarios 3, 5, and 7 that contain ferrite were excluded. Only scenario 1, which also contains ferrite, was kept, why it was adopted as the baseline scenario.

### LCA sensitivity analysis and Ecodesign options proposition

Based on the motor model of the Real product, their respective components were combined and generated eight possible loudspeaker motor design scenarios, as shown in Table 3.

**Table 3:** Combination of components for Ecodesign scenarios.

Possible scenarios	Coil	Coil former	Magnet	Scenarios for Ecodesign
1	Aluminum	Aluminum	Ferrite	$\checkmark$
2	Aluminum	Aluminum	Neodymium	$\checkmark$
3	Aluminum	Fiberglass	Ferrite	
4	Aluminum	Fiberglass	Neodymium	$\checkmark$
5	Copper	Aluminum	Ferrite	
6	Copper	Aluminum	Neodymium	$\checkmark$
7	Copper	Fiberglass	Ferrite	
8	Copper	Fiberglass	Neodymium	$\checkmark$

The combination of the different components of the loudspeaker’s motor was carried out as a Sensitivity Analysis, foreseen as an optional phase of the LCA and also as a proposal of possibilities for the Ecodesign decision-making process.

**Figure 4:**
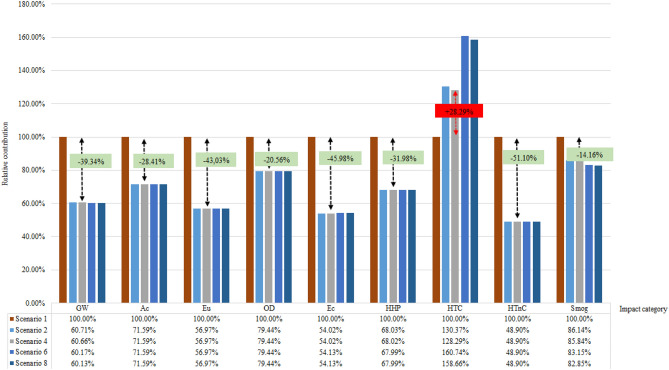
Line graph with two axes and the five scenarios representing the relative contribution in percentage by impact category by categories, highlighted the HTC scenarios 2 and 4 with a percentage of 28.29% above the threshold level of 100%.

In the analysis of Fig. 4, it is observed a homogeneous variation and a standard behavior between the categories of environmental impacts in the projected Ecodesign scenarios. In short, all projected scenarios have better environmental performance than the base scenario (Real product), except for the Human toxicity cancer category, in which all projected scenarios have lower environmental performance than the base scenario. This is a delicate environmental impact category, with special attention to human health and notoriously caused by the neodymium life cycle, corroborating the study reported by^21^. Among the projected Ecodesign scenarios, the one with the highest environmental performance, in absolute terms, is Scenario 4, which is composed of an aluminum coil, fiberglass coil former, and neodymium magnet.

**Figure 5:**
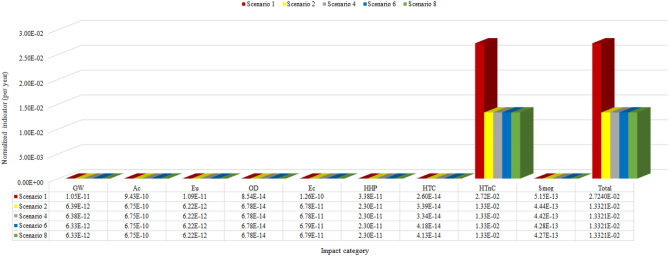
Line chart with two axes and five scenarios representing the normalized indicator per year by category of impact by all categories, being 51% greater than the total of scenario 1 in relation to the others.

On the other hand, to have a view of the environmental performance as a whole, among each evaluated scenario, including the base scenario, external normalization was adopted in relation to the sum of each environmental impact category evaluated in North America, according to data from^32^. Thus, it is possible to have an estimation of the total potential for environmental impacts that each scenario has and compare them with each other. Figure 5 presents these results.

**Figure 6:**
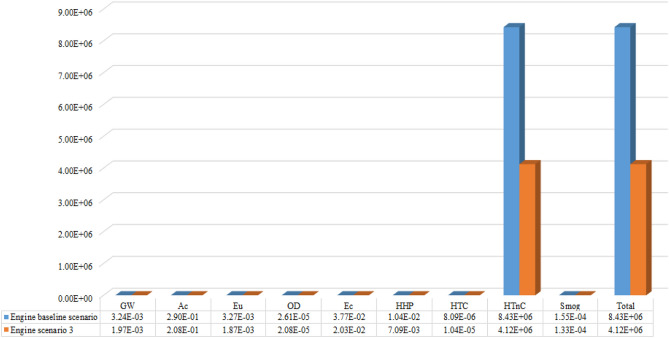
Comparison of the relative contributions by the category indicators, of coil former and coil. The Ec and HTnC category for the coil is highlighted, with its impact percentage being approximately ten times greater than the others.

From the results of external standardization, it is observed that Ecodesign project scenarios 2, 4, 6, and 8 have equivalent environmental performance. Furthermore, despite all of them having higher potential impacts for the Human toxicity non-cancer category, in total, the projected Ecodesign scenarios have relatively 51% less environmental impact potential than the base scenario, which is widely used in the worldwide loudspeaker market. This improvement in environmental performance for all projected scenarios could be a major step forward in the global loudspeaker industry against the Circular Economy, considering the totality of products in the world market. As this is a relative analysis in percentage units, the relative contribution of the Human toxicity non-cancer category is much higher than the other categories, making it difficult to visualize the contributions of the other components and scenarios for the other categories of environmental impacts. However, there is a significant difference, in percentage terms, between the other scenarios depending on the categories of impacts analyzed, as indicated in Fig. 6.

It can be seen that the Ecodesign scenarios that replace aluminum with fiberglass, keeping neodymium in coil former, have superior environmental performance. These are the cases of scenarios 4 and 8. On the other hand, in the same graph, it can be seen that copper presents impact potentials much higher than aluminum in three impact categories. In contrast, the other categories are close to the borderline represented in red and dotted in Fig. 6. Thus, it can be concluded that the Ecodesign 4 scenario for the loudspeaker motor, from the point of view of environmental performance, is the best scenario among the eight evaluated.

## Conclusion

The research objective was successfully completed within the proposed boundaries. Among the eight component replacement scenarios that characterize the Ecodesign proposals, a project specification for the loudspeaker motor with the best-analyzed performance was obtained. The identified proposal is scenario 4, which consists of a motor with an aluminum coil, fiberglass coil former, and neodymium magnet. Furthermore, it was possible to identify the intermediate Ecodesign scenarios for the loudspeaker motor based on the most used motor option in the market.

The results of this article fill a theoretical gap in the scientific literature and contribute to engineering practice with subsidies for the decision-making process in loudspeaker development projects.

The research has two important limitations. The first one is based on data collection for the LCI, which was carried out based on datasheets from the loudspeaker manufacturers, being an approach of collecting data from secondary sources, but with reliability. The second limitation is in the scope of the LCA, which was limited to only one loudspeaker component, the motor, due to the complexity of this product and its production chain.

However, these limitations can be overcome in future research, with the support of manufacturers, adopting data collection from primary sources in the field and in loco and for the loudspeaker as a whole. In addition, there are suggestions for analyzing the technological and economic performance of the product in an integrated manner with the environmental performance. Thus, the results tend to be better absorbed by manufacturers and consumers, and therefore, loudspeaker Ecodesign projects can be implemented on large scales.

## Supplementary Information

Supplementary Information.

## Data Availability

All databases and software used for supporting the conclusion of this article are available at the website of (http://www.gabi-software.com/international/databases/).
