# Supplementary material for: Scenarios for Ecodesign in loudspeaker’s motor
Source: Sci Rep. 2022 Nov 14;12:19493. doi: 10.1038/s41598-022-24042-7 (PMC9663512; doi:10.1038/s41598-022-24042-7)
Supplement: Supplementary file 1 — Supplementary Information. [file 41598_2022_24042_MOESM1_ESM.pdf]

Appendix A Materials Inventory

Table A1 Aluminum coil inventory.

Input			Output		
Resources			***Resources***		
Non-renewable energy sources			*Deposited goods*		
Oil	26.4	kJ	Overload	397	g
Mineral coal	56.2	kJ	Decayed	15.6	g
Natural gas	29	kJ	Waste	2.04	g
Material			Treatable waste	0.0076	g
Non-renewable (elements)			Scraps	87.9	g
Iron	0.567	g	*Emission to air*		
Non-renewable			*Particles*	$4.3\text{x}10^{-7}$	g
Bauxite	224	g	*Heavy metals*	$3.68\text{x}10^{-4}$	g
Chrome ore (39%)	0.151	g	*Inorganic*		
Fluorite CaF_2	1.41	g	Carbon dioxide	332	g
Inert rock	442	g	Carbon monoxide	0.128	g
Limestone	10.5	g	Nitrogen	0.201	g
Potassium (10%, K20)	0.151	g	Nitrogen oxide	0.613	g
Quartz sand (silica)	2.6	g	Sulfur dioxide	0.853	g
Sodium Chloride	5.04	g	*Organic (VOC)*		
Soil	16.6	g	Methane	0.727	g
Clay	3.7	g	NMVOC	0.0699	g
Renewable			*Emissions to water*	221	kg
Water	387.5	g	*Emissions to soil*	$1.23\text{x}10^{-3}$	g
Air	1.4	kg	***Product***		
Carbon Dioxide	4.82	g	Aluminum coil	40.7	g

Table A2 Copper coil inventory.

Input			Output		
Resources			***Resources***		
Non-renewable energy sources			*Deposited goods*		
Oil	21.8	kJ	Overload	8.19	kg
Mineral coal	12.6	kJ	Decayed	2.51	g
Natural gas	7.72	kJ	Waste	4.46	kg
Material			Treatable waste	0.0076	g
Non-renewable (elements)			Scraps	86.8	g
Copper	598	g	*Emission to air*		
Lead	3	g	*Particles*	0.0607	g
Iron	0.155	g	*Heavy metals*	0.0246	g
Zinc	17.5	g	*Inorganic*		
Non-renewable			Carbon dioxide	149	g
Chrome ore (39%)	0.151	g	Carbon monoxide	0.12	g
Fluorite CaF_2	0.267	g	Nitrogen	0.182	g
Inert rock	12.2	kg	Nitrogen oxide	0.357	g
Limestone	5.79	g	Sulfur dioxide	0.734	g
Potassium (10%, K20)	0.306	g	*Organic (VOC)*		
Quartz sand (silica)	10.9	g	Methane	0.192	g
Sodium Chloride	0.938	g	NMVOC	0.0344	g
Soil	6.06	g	*Emissions to water*	145	kg
Clay	3.63	g	*Emissions to soil*	$1.13\text{x}10^{-3}$	g
Renewable			***Product***		
Water	147	kg	Copper coil	40.7	g
Air	903	g			
Carbon Dioxide	7.9	g			

Table A3 Aluminum former inventory.

Input			Output		
Resources			***Resources***		
Non-renewable energy sources			*Deposited goods*		
Oil	1.37	kJ	Overload	24	g
Mineral coal	2.69	kJ	Decayed	0.802	g
Natural gas	1.68	kJ	Waste	0.111	g
Material			Scraps	4.53	g
Non-renewable (elements)			*Emission to air*		
Iron	0.0317	g	*Particles*	7.78x10^{-3}	
Non-renewable			*Heavy metals*	1.87x10^{-5}	g
Bauxite	11.4	g	*Inorganic*		
Inert rock	26.8	g	Carbon dioxide	17.9	
Limestone	0.552	g	Carbon monoxide	7.17x10^{-3}	g
Potassium (10%, K20)	0.0118	g	Nitrogen	1.66x10^{-3}	g
Quartz sand (silica)	0.135	g	Nitrogen oxide	0.0326	g
Sodium Chloride	0.238	g	Sulfur dioxide	0.0435	g
Soil	0.852	g	*Organic (VOC)*		
Clay	0.191	g	Methane	0.039	
Renewable			NMVOC	3.78x10^{-3}	g
Water	184	kg	*Emissions to water*	185	kg
Air	77.5		*Emissions to soil*	2.8x10^{-5}	g
Carbon Dioxide	0.393	g	***Product***		
			Aluminum former	2.07	g

Table A4 Fiberglass former inventory.

Input			Output		
Resources			***Resources***		
Non-renewable energy sources			*Deposited goods*		
Oil	0.0618	kJ	Overload	10.3	g
Mineral coal	0.202	kJ	Decayed	0.027	g
Natural gas	0.714	kJ	Waste	0.14	g
Material			Scraps	0.446	g
Non-renewable (elements)			*Emission to air*		
Iron	4.18x10^{-3}	g	*Particles*	9.73x10^{-4}	g
Non-renewable			*Heavy metals*	2x10^{-6}	g
Colemanite ore	0.273	g	*Inorganic*		
Dolomite $CaMg_2CO_3$	0.179	g	Carbon dioxide	3.39	g
Inert rock	11.2	g	Carbon monoxide	1.25x10^{-3}	g
Limestone	0.856	g	Nitrogen	3.77x10^{-4}	g
Potassium (10%, K20)	0.011	g	Nitrogen oxide	2.28ex10^{-3}	g
Quartz sand (silica)	0.844	g	Sulfur dioxide	0.0163	g
Soil	0.867	g	*Organic (VOC)*		
Clay	0.0185	g	Methane	5.75x10^{-3}	g
Renewable			NMVOC	1.09x10^{-3}	g
Water	3.89	kg	*Emissions to water*	3.97	kg
Air	13.9	g	*Emissions to soil*	2.1x10^{-5}	g
Carbon Dioxide	0.346	g	***Product***		
			Glassfiber former	2.07	g
